# The Patency Rate of the Primary and Exchanged Femoral Haemodialysis Catheters

**DOI:** 10.21315/mjms2021.28.4.10

**Published:** 2021-08-26

**Authors:** Roozbeh Cheraghali, Pezhman Farshidmehr

**Affiliations:** 1 Vascular and Endovascular Surgery, Golestan University of Medical Sciences, Gorgan, Iran; 2 Vascular and Endovascular Surgery, Tehran University of Medical Sciences, Tehran, Iran

**Keywords:** tunneled femoral catheter, patency rate, survival, complication, mortality

## Abstract

**Background:**

This prospective cohort study aims to evaluate the primary and exchanged femoral catheter patency rates, as well as mortality rates and determine the probable risk factors affecting femoral catheter survival.

**Methods:**

All 79 tunneled femoral catheters created in our hospital from 2017 to 2020 were included in this study. Patients having no other means for dialysis access other than the femoral catheter was recruited in this study. Data collected included patient age, sex, comorbidities (diabetes and hypertension), transplant history, dialysis duration, catheter complications, femoral access history, and primary and exchanged femoral patency rates. Patients were followed for 4–36 months.

**Results:**

The median catheter primary patency was 7 months (95% confidence interval [CI]: 5.77, 8.22) and the primary patency rates at 2, 4 and 6 months were 79%, 68% and 48%, respectively. The median exchanged catheter survival was 8 months (95% CI: 0.83, 15.17) and the exchanged patency rates at 1, 3 and 8 months were 72%, 64% and 32%, respectively. Of the patients (*n* = 62), 8% (5 patients) died because they had no other option for dialysis access.

**Conclusion:**

Tunneled femoral catheters have a low patency rate and should be the last option for haemodialysis patients when other probable accesses are not available.

## Introduction

Vascular access is a crucial requirement when performing haemodialysis. Vascular access can be of different types: arteriovenous fistulae (AVF), arteriovenous synthetic grafts and deep vein (internal jugular, subclavian, translumbar, and femoral) catheterisation (1–2). The femoral vein is the choice when the prolonged use of the upper extremities leads to the occlusion of the central veins (3). However, tunneled femoral vein dialysis catheters for long-term use are associated with poor access patency and increased complication rates, including catheter-related infection and deep vein thrombosis (DVT). Prolonging catheter patency and preventing complications in these patients are essential (4). This prospective cohort study aims to evaluate the primary and exchanged femoral catheter patency rates, as well as mortality rates, and determine the probable risk factors that may affect femoral catheter survival.

## Methods

This prospective cohort study was conducted in a single referral hospital from July 2017 to September 2020. We recruited patients that had no other means for dialysis access other than the femoral catheter. Between 2017 and 2020, all 79 tunneled femoral catheters (census sampling) were included in this study. We performed all procedures in our vascular access centre. From July 2017 to September 2020 (38 months), one of us, an experienced vascular surgeon, placed all tunneled femoral catheters in 62 patients. During the follow-up, 17 patients required a catheter exchange. After obtaining informed consent, a vascular surgeon placed catheters in patients under local anesthesia with 2% lidocaine in the operating room. We used real-time ultrasound and fluoroscopic guidance in the fluoroscopy suite, and 31 cm–55 cm catheters were used. The tip of the catheter was placed in the proximal inferior vena cava (IVC). The skin exit site for the tunneled femoral catheters was in the lateral thigh, 5 cm–7 cm inferior to the iliofemoral line. We performed a follow-up for outcomes and complications in all patients and maintained a database of all procedures. Data collected included patient age, sex, diabetes, hypertension, transplant history, dialysis duration, catheter complications, femoral access history, and primary and exchanged femoral patency rates. The exchanged patency rate was separately calculated for all exchanged catheters. Institutional review board approval was obtained.

In the dialysis unit, all patients and medical personnel were required to wear a gown, mask and cap. The medical personnel covered hubs and catheters with swabs soaked with 2% chlorhexidine during connection and disconnection, and used transparent, semipermeable polyurethane dressings to cover the catheter exit site to allow visual inspection. Nurses exchanged dressing every week and replaced dressings, if necessary, three times a week. Heparin (1,000 μ/mL) sealed all catheters in our units; we did not use citrate or antibiotic locks. In all cases, we followed the same precautions to handle the femoral catheters and changed dressings after each haemodialysis session. All patients were dialysed using Fresenius Machines 4008 S, three times per week, for 3 h–4 h, with high-flow, high-efficiency and high-permeability polysulfone dialysers.

For catheters in which adequate flows were not obtained (200 mL/min), catheter clearance was tried with tissue plasminogen activator up to a maximum of three times before the catheter was considered nonfunctional. If the function was not restored, the catheter was exchanged.

Catheter exchanges were performed by passing a stiff guide wire through one lumen of the catheter. After contrast injection for venography, if fibrin sheath was observed, it was disrupted with balloon angioplasty; then, a new catheter was inserted through the guide wire.

The primary patency rate was defined as the number of catheter days from the time of catheter creation to the removal at the end of therapy, patient death, catheter exchange or removal (event). We also report the exchanged patency rate, which is the number of functional days from the time of the catheter exchange to catheter failure or removal (event). Censored data are those femoral catheters that are functional after the study period.

## Statistical Analysis

We expressed values as median, mean ± standard deviation (SD) or percentage as appropriate. The Wilcoxon rank-sum test for continuous variables (e.g. age and dialysis duration) and Chi-square or Fisher’s exact tests for categorical variables were used. We generated survival curves for primary catheter patency with Kaplan-Meier methodology and differences between groups with the log-rank test. SPSS version 16 was used for all analysis and *P*-value of less than 0.05 was considered statistically significant.

## Results

A total of 79 tunneled femoral catheter insertions were included in the study, of which 17 were exchanged catheters. Demographic and catheter information are summarised in Table 1. Of the 62 patients, 33 were males and 29 were females. The mean age of patients and dialysis duration were 54.5 (range 21–87) and 5.03 years, respectively (SD = 3.75). The two patient groups (functional versus failed catheters during the study period) differed with respect to mean age, comorbidities (diabetes and hypertension) and history of previous femoral access in that site. The two groups were similar in sex, transplant history, starting access for dialysis, complications and dialysis duration time.

**Figure 1:**
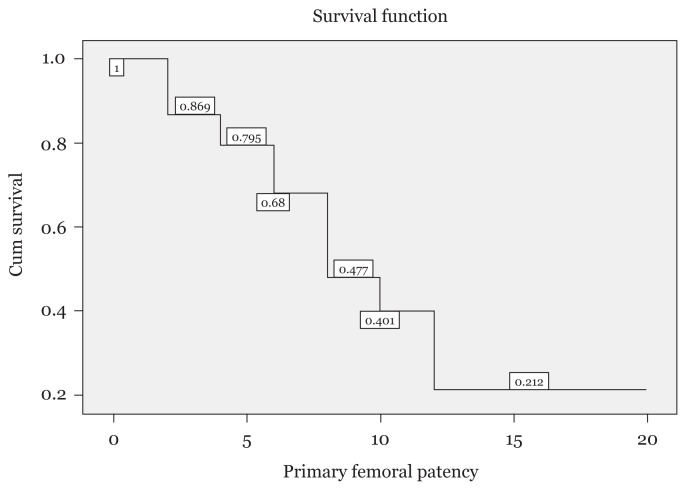
Survival function of the primary patency rate of femoral haemodialysis catheters

The Kaplan-Meier analysis revealed that the median primary patency was 7 months (95% confidence interval [CI]: 5.77, 8.22) and that the primary patency rates at 2, 4 and 6 months were 79%, 68% and 48%, respectively (life table analysis). Figure 1 summarises the survival function of the primary patency rate.

**Figure 2:**
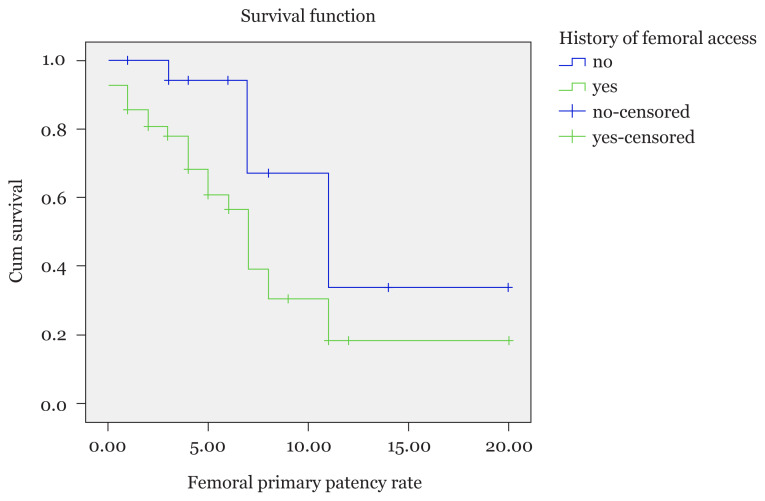
Survival function of the primary patency rate based on the history of previous femoral access

The median exchanged catheter survival was 8 months (95% CI: 0.83, 15.17) and the exchanged patency rates at 1, 3 and 8 months were 72%, 64% and 32%, respectively. The difference of the median survival of the primary patency time based on the history of previous femoral access was statistically significant (*P*-value = 0.019; Figure 2).

The mean follow-up time was 20 months (range 4–36 months) and 25 deaths occurred during this period. Of the patients, 8% died because of having no other means for haemodialysis (Table 2). DVT and infection occurred in 32 (51.6%) and 13 (21%) catheters, respectively. DVT occurred in the ipsilateral lower extremity. All patients presented with unilateral lower extremity edema within two weeks after catheter placement and were confirmed by ultrasonography. Because of the absence of other options for vascular access, all patients with DVT were treated with anticoagulation therapy and dialysis was continued without the removal of the catheter. No patient had a bleeding complication related to the anticoagulation.

In Cox regression analysis, age, sex, history of femoral catheter and comorbidities (diabetes and hypertension) were in the equation. Older age (hazard ratio [HR]: 1.03) and comorbidities (diabetes and hypertension; HR: 1.08) affect the primary patency rate of femoral catheters (*P*-value = 0.032 and 0.049, respectively) (Table 3).

## Discussion

Despite the use of the femoral vein as a site for dialysis catheter insertion, the overall patency of catheters in this location is thought to be poorer than those in the upper extremities.

In this series, the mean primary patency was 5.4 months compared with the mean patency of 51 days in a study by Maya and Allon (5). In another study by Falk (6), the median patency was 59 days and it was 61 days in a study by Zaleski et al. (7). When comparing primary patency, we observed 88% at 1 month, whereas Burton et al. (8) observed 54% and Zaleski et al. (7) observed 78% at 1 month. Exchanged femoral catheters had poorer survival results than the primary patency rate, as all of them had a history of recent femoral catheterisation, which can affect their patency rate. In a case series, the functional life of the femoral catheter ranges from 2.5 to 19.5 months (1). This difference may be the outcome of better nursery care at dialysis units, creating catheters by an experienced hand, various catheter lengths, or better inpatient or outpatient care after catheter insertions.

We evaluated the factors that may affect the patency rate; older age, history of previous femoral access and cause of end-stage renal disease (ESRD) affected the mean patency of femoral accesses. We did not examine the right or left site of the catheters, although there was no statistical difference in the patency rate of the left or right site of the catheters according to Burton et al. (8). The right femoral vein insertion site may be preferable than the left-sided insertions because the route to the IVC from the right iliac veins is short and straight relative to those on the left (6).

Older age and diabetes can increase the risk of catheter failure because patients with diabetes are susceptible to infection that may lead to catheter failure (8).

Our catheter failure rate was 48.4%, and it was 30.1% and 27% in the studies of Burton et al. and Falk, respectively. This difference may come from our longer follow-up period (6–8).

Sepas et al. (9) reported 28.6% DVT and 23.2% infection of the femoral catheters. In this series, we had a similar infection rate (21%) but DVT was detected in half of the patients (50.6%).

More studies are needed to determine the potential causes of femoral catheter infection or thrombosis, which may cause DVT and its consequent complications.

In Shindo et al. (10)’s research, no factors, including age, sex, body mass index, dialysis condition, comorbidities and medications, were significantly associated with catheter survival. In another study, the previous history of femoral catheterisation was the only factor that affected the mean patency time (9). They also had a higher infection rate in females than in males; however, in this study, the infection rate did not statistically differ between sex groups (*P*-value = 0.82). According to our data, older age and risk factors of ESRD (diabetes, hypertension and renal stone) affect the survival of femoral catheters. Although the mean patency time was statistically different between two groups of patients with or without a history of previous femoral catheterisation, it was not a predictive factor in Cox regression analysis when evaluated with other factors. Larger studies are essential to evaluate the predictive factors to omit confounding factors.

## Conclusion

We recommend physicians create an AVF in patients with ESRD as soon as possible to prevent repeated central vein catheterisations as they lead to central vein occlusion and creating AVFs will be more difficult later. Tunneled femoral catheters have a low patency rate and should be the last option for haemodialysis patients when other probable accesses are not available. Increasing the knowledge of patients on how to maintain their accesses to improve the patency rate is also crucial.

## Figures and Tables

**Table 1:** Demographic and catheter information

		Failure: *n* = 30 Frequency (%)	Functional: *n* = 32 Frequency (%)	*P*-value
Male		14 (46.7)	19 (59.4)	0.316
Female		16 (53.3)	13 (40.6)	
History of femoral access		24 (80.0)	19 (59.0)	0.046^1^
Diabetes		4 (13.3)	7 (21.9)	0.0431
Hypertension		6 (20.0)	13 (40.6)	
History of transplant	No	26 (86.7)	28 (87.5)	0.922
	Yes	4 (13.3)	4 (12.5)	
Start access	Tem cath	29 (96.7)	25 (78.1)	0.101
	Perm cath	-	3 (9.4)	
	AVF	-	3 (9.4)	
	Transplant	1 (3.3)	1 (3.1)	
Complications	DVT	16 (53.3)	16 (50.0)	0.364
	Infection	8 (26.7)	5 (5.6)	
	Others	6 (20.0)	DVT + infection:	
			2 (6.2)	
			9 (28.1)	
Age^3^		58.76 (1.68)	50.62 (1.36)	0.040^2^
Dialysis duration^3^ (year)		5.63 (3.05)	4.46 (4.28)	0.225
Primary femoral patency^3^		4.93 (3.60)	5.93 (4.85)	0.366
Exchanged femoral patency^3^		2.00 (2.82)	4.36 (2.97)	0.114

Notes:

1 Chi-square test;

2 independent sample *t*-test;

3 mean (SD)

**Table 2:** Causes of mortality among the study participants

Mortality cause	Number (%)
Not related to catheter	11 (17.7)
Related to catheter	3 (4.8)
No access	5 (8.1)

**Table 3:** Cox regression analysis: Factors affecting the primary patency

Variables in the equation	β	SE	Sig.	Exp (β)	95% CI for Exp (β)	

					Lower	Upper
Age	0.032	0.015	0.032	1.032	1.003	1.063
Comorbidities (diabetes and hypertension)	0.078	0.040	0.049	1.082	1.000	1.169
Sex	0.631	0.393	0.108	1.880	0.870	4.061
History of femoral access	0.519	0.515	0.313	1.681	0.613	4.609

Notes: β = beta coefficient; SE = standard error; Sig. = *P*-value; Exp (β) = odds ratio; 95% CI for Exp (β) = 95% CI odds ratio
